# Equilibrium model selection: dTTP induced R1 dimerization

**DOI:** 10.1186/1752-0509-2-15

**Published:** 2008-02-04

**Authors:** Tomas Radivoyevitch

**Affiliations:** 1 Department of Epidemiology and Biostatistics, Case Western Reserve University, 10900 Euclid Avenue, Cleveland, OH 44106, USA

## Abstract

**Background:**

Biochemical equilibria are usually modeled iteratively: given one or a few fitted models, if there is a lack of fit or over fitting, a new model with additional or fewer parameters is then fitted, and the process is repeated. The problem with this approach is that different analysts can propose and select different models and thus extract different binding parameter estimates from the same data. An alternative is to first generate a comprehensive standardized list of plausible models, and to then fit them exhaustively, or semi-exhaustively.

**Results:**

A framework is presented in which equilibriums are modeled as pairs (*g*, *h*) where *g *= 0 maps total reactant concentrations (system inputs) into free reactant concentrations (system states) which *h *then maps into expected values of measurements (system outputs). By letting dissociation constants *K*_*d *_be either freely estimated, infinity, zero, or equal to other *K*_*d*_, and by letting undamaged protein fractions be either freely estimated or 1, many *g *models are formed. A standard space of *g *models for ligand-induced protein dimerization equilibria is given. Coupled to an *h *model, the resulting (*g*, *h*) were fitted to dTTP induced R1 dimerization data (R1 is the large subunit of ribonucleotide reductase). Models with the fewest parameters were fitted first. Thereafter, upon fitting a batch, the next batch of models (with one more parameter) was fitted only if the current batch yielded a model that was better (based on the Akaike Information Criterion) than the best model in the previous batch (with one less parameter). Within batches models were fitted in parallel. This semi-exhaustive approach yielded the same best models as an exhaustive model space fit, but in approximately one-fifth the time.

**Conclusion:**

Comprehensive model space based biochemical equilibrium model selection methods are realizable. Their significance to systems biology as mappings of data into mathematical models warrants their development.

## Background

Ribonucleotide reductase (RNR) has a small subunit R2 that exists almost exclusively as a dimer, and a large subunit R1 that dimerizes when dTTP, dGTP, dATP, or ATP binds to its specificity site, and hexamerizes when dATP or ATP binds to its activity site [1-6]. Thus, R1 is the backbone of a biochemical equilibrium network that contains a large number of R1 complexes. This network has more dissociation constants (*K*_*d*_) than can be estimated from currently available data, so assumptions must be made to reduce the number of independent *K*_*d*_. These assumptions come in two forms: those that state that for the data at hand, a *K*_*d *_is too large or small to be distinguished from infinity or zero, respectively, and those that state that the data are too weak to rule out a null hypothesis of the form *K*_*d *_= ${K^{\prime }}_{d}$. Model parameters such as the fraction of R1 capable of forming dimers and hexamers, and the enzymatic activities of these R1 states, also come with plausible null hypotheses. In general, different null hypotheses define different models that yield different estimates of the freely estimated parameters. Unfortunately, as modelers traverse a path of reasonable hypotheses until they arrive at a model that provides both a good fit and *K*_*d *_confidence interval limits that are not too wide, they often stop at different places, and thus report different *K*_*d *_values. Such *K*_*d *_estimate extraction differences could be reduced, if a systematic reproducible approach to biochemical equilibria model building was established. Progress toward this goal is described in this paper.

## Results

### Model

Consider a dataset comprised of *N *steady state non-covalent binding equilibriums indexed by *n *in which *J *different complexes can potentially form from a protein R of known total concentration *T*_*n*1 _through interactions with itself and *I *- 1 other reactants (e.g. substrate, effectors and other proteins) of known total concentrations *T*_*ni *_(1 <*i *≤ *I*). Suppose *W*_*ij *_copies of the *i*th reactant exist in the *j*th complex and that a particular R molecule is either undamaged with probability *p*, and thus capable of forming each of the plausible complexes, or damaged with probability 1 - *p*, and thus incapable of forming any complexes. Define *T*_*n *_= (*T*_*n*1_, *T*_*n*2_, ...*T*_*nI*_), *F*_*n *_= (*F*_*n*1_, *F*_*n*2_, ...*F*_*nI*_) as the corresponding free reactant concentrations, *K *= (*K*_1_, *K*_2_, ...*K*_*J*_) as the dissociation constants (of complexes to free reactants), *y*_*n *_as the measurement(s) made at the *n*th steady state, and *Z*_*n *_= (*Z*_*n*1_, *Z*_*n*2_, ...*Z*_*nJ*_) as the concentrations of complexes predicted by *W*, *K *and *F*_*n *_to be

$$Z_{nj}=\frac{\prod_{i^{\prime }=1}^{I}F_{ni^{\prime }}^{W_{i^{\prime }j}}}{K_{j}}.$$

The relationship between the system inputs (*T*_*n*_), states (*F*_*n*_) and outputs (*y*_*n*_) is then modeled by *I *total concentration constraints

*g*(*F*_*n*_, *T*_*n*_, *K*, *p*) = 0

that must be solved for the *I *free reactant concentrations *F*_*n *_at each *n *(1 <*n *≤ *N*) given the inputs *T*_*n*_, and an output measurement model *h *that connects *F*_*n *_to expected values of the outputs *E*(*y*_*n*_)

*y*_*n *_= *h*(*F*_*n*_, *K*, *p*, *L*) + *ε*_*n*_

where all of the *h *specific parameters (e.g. *k*_*cat*_'s and protein masses) are contained in the vector *L *and, if the *y*_*n *_are vectors of measurements, the *e*_*n *_are vectors of zero mean noise, potentially correlated within steady states, but uncorrelated between steady states; only scalar *y*_*n *_are considered hereafter. The model parameters *K*, *p *and *L *are not indexed by *n *because they are fitted jointly to the entire dataset, i.e. one set of estimates of these parameters describes all *N *steady states simultaneously as one (*g*, *h*) model of one underlying biochemical equilibrium network.

### System models

The *I *equations of a system model *g *= 0 are

$$\begin{array}{c} g_{1}(F_{n},T_{n},K,p)=pT_{n1}-F_{n1}\\ -\sum_{j=1}^{J}W_{1j}\frac{\prod_{i^{\prime }=1}^{I}F_{ni^{\prime }}^{W_{i^{\prime }j}}}{K_{j}}\\ =0\\ g_{i}(F_{n},T_{n},K)=T_{ni}-F_{ni}\\ -\sum_{j=1}^{J}W_{ij}\frac{\prod_{i^{\prime }=1}^{I}F_{ni^{\prime }}^{W_{i^{\prime }j}}}{K_{j}}\\ \begin{array}{cc} =0 & \left(1<i\leq I\right) \end{array} \end{array}$$

where *pT*_*n*1 _is the total concentration of undamaged R and *F*_*n*1 _is the concentration of free R that is undamaged and thus capable of forming complexes. If all biologically plausible candidate complexes are present in these equations, the model will have as many *K *parameters as possible, and it will therefore be called a full model. A space of *g *= 0 models can then be generated from this full model through combinations of null hypothesis constraints on the parameters in (*K*, *p*).

Fitting a particular (*g*, *h*) to data (*T*, *y*) to estimate parameters in (*K*, *p*, *L*) demands many repeated solutions of *g *= 0. These equations must be solved efficiently to fit large model spaces and models with large numbers of parameters. The approach proposed here solves *g *= 0 by letting *g *be the right hand side of a parent set of ordinary differential equations (ODEs) that achieves *g *= 0 at steady state. Specifically, the following ODEs were simulated to large Τ to solve the polynomial system in Eqs. (2):

$$\begin{array}{lll} \frac{dF_{n1}}{d\tau } & = & pT_{n1}-F_{n1}-\sum_{j=1}^{J}W_{1j}\frac{\prod_{i^{\prime }=1}^{I}F_{ni^{\prime }}^{W_{i^{\prime }j}}}{K_{j}}\\ \frac{dF_{ni}}{d\tau } & = & T_{ni}-F_{ni}-\sum_{j=1}^{J}W_{ij}\frac{\prod_{i^{\prime }=1}^{I}F_{ni^{\prime }}^{W_{i^{\prime }j}}}{K_{j}} \end{array}$$

where 1 <*i *≤ *I*, *n *= 1...*N *and *F*_*ni*_(0) = 0. Note that the initial conditions guarantee that the system derivatives are initially positive and thus that the system always starts in an acceptable direction; model parameters are constrained to positive values, expressed internally as *e*^*c*^, where *c *is unconstrained during optimization.

The system of polynomials in Eqs. (2) has been solved by others using other approaches. In one approach, the *F*_*ni *_terms are pulled to the left hand side and guesses are then iteratively entered into the right hand side until the equations become self consistent [7]. This approach has more recently been shown to fail in cases of oligomerization, and modifications of the approach have been suggested [8]. The difficulties of solving systems of arbitrary nonlinear algebraic equations in general have been described [9] and a common approach (e.g. used by fsolve in Matlab) has been to minimize the sum of squares *g*^2 ^using Levenberg-Marquadt or Gauss-Newton methods. Intuitively, methods that exploit the fact that the equations are strictly polynomials should outperform these general methods. Continuation homotopy is one such method [10]. In this method, polynomials are homogenized to a larger polynomial system with known solutions, and these solutions are then traced to the desired solutions as the homogenized polynomials are continuously morphed back to the original polynomial system. On a practical level, all complex initial solutions must be tracked to find the desired final solution that is strictly real and positive, and this makes the approach slower than the R [11] implementation of Eqs. (3) provided here, which finds only the positive real root and does so rapidly because it automatically generates and compiles C code (of Eqs. 3) that is then used with the dll/so option of the ODE solver lsoda available in R [11]. To glean some insight into why Eq. (3) works, note that the *g*_*i *_(i.e. right hand sides) are all initially positive, and all monotonically decreasing functions of increasing free concentrations. Free concentration differentials thus start positive and shrink toward zero as the free concentrations move out of their initial values at the origin and into the positive quadrant. When a component *F*_*ni *_of the vector *F*_*n *_crosses its steady state value, the corresponding *g*_*i *_switches signs, since the *g*_*i *_continue to decrease monotonically through zero, and *F*_*ni *_is then thus driven back toward a smaller value, i.e. back toward the steady state value that it just crossed. This explains why the proposed algorithm is stable. Finally, an alternative approach to the problem is to solve *g *= 0 using full-blown kinetic equations with irrelevant time scales defined by *k*_*on *_= 1 and *k*_*off *_= *K*_*d*_, but the number of ODEs then equals the number of complex species plus the number of reactants, rather than just the number of reactants as in Eqs. 3, and although each ODE is computationally simpler in this case, the savings per ODE do not offset the added cost of the additional ODEs. This added cost is expected to become substantial if not prohibitive in combinatorially complex scenarios wherein the number of complexes is very large relative to the number of reactants.

### *K *hypotheses

In the *g *= 0 model in Eqs. (2), the elements of *K *are defined as

$$K_{j}=\frac{\prod_{i^{\prime }=1}^{I}F_{ni^{\prime }}^{W_{i^{\prime }j}}}{Z_{nj}}.$$

**Figure 1:**
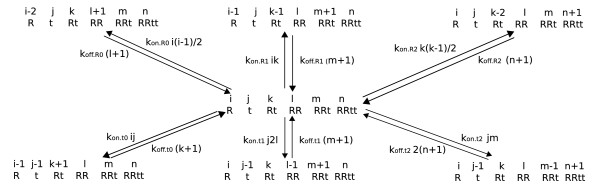
**Rt system state transition diagram**. The next states of a unit volume reaction vessel that currently has (*i*, *j*, *k*, *l*, *m*, *n*) molecules of (R, t, Rt, RR, RRt, RRtt) are shown. The *k*_*on*_'s in this diagram are the rates at which potential interactions successfully materialize, and the *k*_*off *_'s are the per-site rates at which ligands dissociate.

This definition can differ by stoichiometric factors from *K*_*d *_defined as *k*_*off*_/*k*_*on*_. For example, consider a system where R can bind a ligand t and R can also form dimers. Figure 1 shows the state transitions of this system from a state of *i*, *j*, *k*, *l*, *m *and *n *molecules of R, t, Rt, RR, RRt and RRtt, respectively, per unit volume, where the unit volume is small enough that any reactant can react equally well with any other reactant, yet large enough that these integers are approximately equal to themselves plus or minus one or two. If net fluxes between states are zero, the system is in equilibrium and the following definitions of *K*_*d *_≡ *k*_*off*_/*k*_*on *_arise

$$\begin{array}{lll} k_{on.R0}i(i-1)/2 & = & \begin{array}{cc} k_{off.R0}(l+1) & \Rightarrow \end{array}\\ K_{d\_R\_R} & \equiv & k_{off.R0}/k_{on.R0}\\ & = & \frac{i(i-1)}{2(l+1)}\approx \frac{\left[R][R\right]}{2[RR]} \end{array}$$

$$\begin{array}{lll} k_{on.R1}ik & = & \begin{array}{cc} k_{off.R1}(m+1) & \Rightarrow \end{array}\\ K_{d\_Rt\_R} & \equiv & k_{off.R1}/k_{on.R1}\\ & = & \frac{ik}{\left(m+1\right)}\approx \frac{\left[Rt][R\right]}{\left[RRt\right]} \end{array}$$

$$\begin{array}{lll} k_{on.R2}k(k-1)/2 & = & \begin{array}{cc} k_{off.R2}(n+1) & \Rightarrow \end{array}\\ K_{d\_Rt\_Rt} & \equiv & k_{off.R2}/k_{on.R2}\\ & = & \frac{k(k-1)}{2(n+1)}\approx \frac{\left[Rt][Rt\right]}{2[RRtt]} \end{array}$$

$$\begin{array}{lll} k_{on.t0}ij & = & \begin{array}{cc} k_{off.t0}(k+1) & \Rightarrow \end{array}\\ K_{d\_R\_t} & \equiv & k_{off.t0}/k_{on.t0}\\ & = & \frac{ij}{k+1}\approx \frac{\left[R][t\right]}{\left[Rt\right]} \end{array}$$

$$\begin{array}{lll} k_{on.t1}j2l & = & \begin{array}{cc} k_{off.t1}(m+1) & \Rightarrow \end{array}\\ K_{d\_RR\_t} & \equiv & k_{off.t1}/k_{on.t1}\\ & = & \frac{j2l}{m+1}\approx \frac{2[RR][t]}{\left[RRt\right]} \end{array}$$

$$\begin{array}{lll} k_{on.t2}jm & = & \begin{array}{cc} k_{off.t2}2(n+1) & \Rightarrow \end{array}\\ K_{d\_RRt\_t} & \equiv & k_{off.t2}/k_{on.t2}\\ & = & \frac{jm}{2(n+1)}\approx \frac{\left[RRt][t\right]}{2[RRtt]}. \end{array}$$

In Eqs. 5 and 7, *x*(*x *- 1)/2 is the number of unique binary interactions of *x *molecules with themselves. The stoichiometric factor in Eq. (9) arises because RR has twice as many ways to gain a t as RRt has ways to lose a t, and in Eq. 10 it arises because RRtt has twice as many ways to lose a t as RRt has ways to gain a t. Eqs. 9 and 10 assume that RR and RRtt are symmetric dimers.

**Figure 2:**
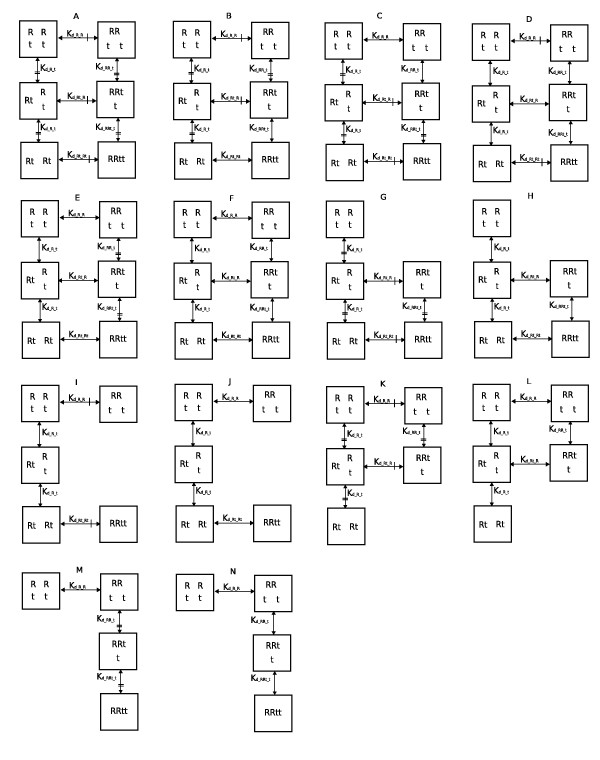
**Space of *K*_*d *_equivalence grid graph models**. In these *K*_*d *_= ${K^{\prime }}_{d}$ grid graphs t dimension edges marked = are equal and R dimension edges marked | are equal, i.e. Model A is fully constrained. Models F, H, J, L and N have zero *K*_*d *_equivalence constraints and are thus equal to Models A-E in Figure 3.

**Figure 3:**
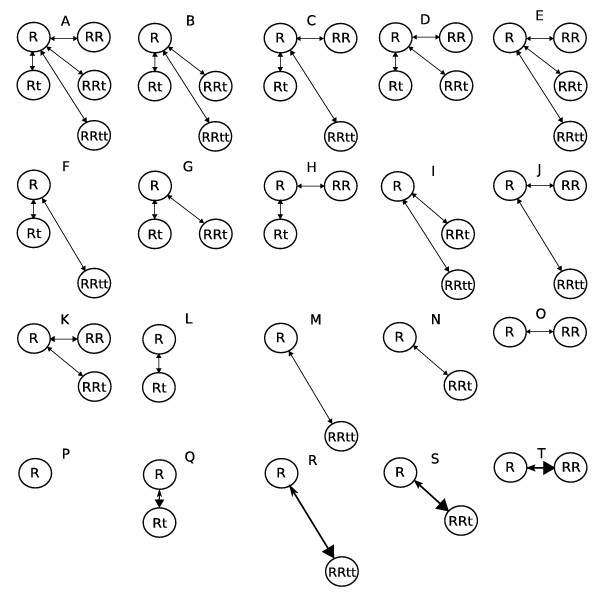
**Space of *K*_*j *_= ∞ or 0 spur graph models**. The full spur graph in A spawns this *g *space of system models. Models Q to T correspond to infinitely tight binding. Models I, J, M, N, R and S cannot be represented by grid graphs.

Regarding differences between the *K*_*d *_in Eqs. (5–10) and the *K*_*j *_in Eq. (4), the *K*_*d *_always have units of concentration because they always correspond to two molecules binding together at one time, and the *K*_*j *_have units of concentrations raised to integer powers $\sum_{i^{\prime }=1}^{I}W_{i^{\prime }j}-1$ that can be greater than 1 (in such cases the *K*_*j *_represent several sequential binding steps condensed into one, e.g. see Table 1). In general, the *K*_*d *_are associated with grid-shaped equilibrium network graphs such as those shown in Figure 2 and the *K*_*j *_are associated with spur-shaped equilibrium graphs such as those shown in Figure 3. Notationally, subscripts of the *K*_*j *_will be distinguishably devoid of *d*'s and underscores, e.g. $K_{RRtt}=\frac{{\left[R\right]}^{2}{\left[t\right]}^{2}}{\left[RRtt\right]}$ is the *K*_*j *_of graph M in Figure 3.

**Table 1:** *K*_*j *_assignment model definitions

graph	*K*_*Rt*_	*K*_*RR*_	*K*_*RRt*_	*K*_*RRtt*_
2A	*K*_*d_R_t*_	2*K*_*d_R_R*_	*K*_*d_R_t*_*K*_*d_R_R*_	$2K_{d\_R\_t}^{2}K_{d\_R\_R}$
2B	*K*_*d_R_t*_	2*K*_*d_R_R*_	*K*_*d_R_t*_*K*_*d_R_R*_	$2K_{d\_R\_t}^{2}K_{d\_Rt\_Rt}$
2C	*K*_*d_R_t*_	2*K*_*d_R_R*_	*K*_*d_R_t*_*K*_*d_Rt_Rt*_	$2K_{d\_R\_t}^{2}K_{d\_Rt\_Rt}$
2D	*K*_*d_R_t*_	2*K*_*d_R_R*_	*K*_*d_R_t*_*K*_*d_Rt_R*_	$2K_{d\_R\_t}^{2}K_{d\_R\_R}$
2E	*K*_*d_R_t*_	2*K*_*d_R_R*_	*K*_*d_R_R*_*K*_*d_RR_t*_	$2K_{d\_R\_R}K_{d\_RR\_t}^{2}$
3A, 2F	*K*_*d_R_t*_	2*K*_*d_R_R*_	*K*_*d_R_t*_*K*_*d_Rt_R*_	$2K_{d\_R\_t}^{2}K_{d\_Rt\_Rt}$
3A, 2F	*K*_*d_R_t*_	2*K*_*d_R_R*_	*K*_*d_R_R*_*K*_*d_RR_t*_	2*K*_*d_R_R*_*K*_*d_RR_t*_*K*_*d_RRt_t*_
2G	*K*_*d_R_t*_	∞	*K*_*d_R_t*_*K*_*d_Rt_R*_	$2K_{d\_R\_t}^{2}K_{d\_Rt\_R}$
3B, 2H	*K*_*d_R_t*_	∞	*K*_*d_R_t*_*K*_*d_Rt_R*_	$2K_{d\_R\_t}^{2}K_{d\_Rt\_Rt}$
2I	*K*_*d_R_t*_	2*K*_*d_R_R*_	∞	$2K_{d\_R\_t}^{2}K_{d\_R\_R}$
3C, 2J	*K*_*d_R_t*_	2*K*_*d_R_R*_	∞	$2K_{d\_R\_t}^{2}K_{d\_Rt\_Rt}$
2K	*K*_*d_R_t*_	2*K*_*d_R_R*_	*K*_*d_R_t*_*K*_*d_R_R*_	∞
3D, 2L	*K*_*d_R_t*_	2*K*_*d_R_R*_	*K*_*d_R_t*_*K*_*d_Rt_R*_	∞
2M	∞	2*K*_*d_R_R*_	*K*_*d_R_R*_*K*_*d_RR_t*_	$2K_{d\_R\_R}K_{d\_RR\_t}^{2}$
3E, 2N	∞	2*K*_*d_R_R*_	*K*_*d_R_R*_*K*_*d_RR_t*_	2*K*_*d_R_R*_*K*_*d_RR_t*_*K*_*d_RRt_t*_
3F	*K*_*d_R_t*_	∞	∞	$2K_{d\_R\_t}^{2}K_{d\_Rt\_Rt}$
3G	*K*_*d_R_t*_	∞	*K*_*d_R_t*_*K*_*d_Rt_R*_	∞
3H	*K*_*d_R_t*_	2*K*_*d_R_R*_	∞	∞
3I*	∞	∞	*K*_*d_R_t*_*K*_*d_Rt_R*_	$2K_{d\_R\_t}^{2}K_{d\_Rt\_Rt}$
3J*	∞	2*K*_*d_R_R*_	∞	$2K_{d\_R\_t}^{2}K_{d\_Rt\_Rt}$
3K*	∞	2*K*_*d_R_R*_	*K*_*d_R_t*_*K*_*d_Rt_R*_	∞
3L	*K*_*d_R_t*_	∞	∞	∞
3M*	∞	∞	∞	$2K_{d\_R\_t}^{2}K_{d\_Rt\_Rt}$
3N*	∞	∞	*K*_*d_R_t*_*K*_*d_Rt_R*_	∞
3O	∞	2*K*_*d_R_R*_	∞	∞
3P	∞	∞	∞	∞
3Q	0	∞	∞	∞
3R	∞	∞	∞	0
3S	∞	∞	0	∞
3T	∞	0	∞	∞

*K_*d_R_t *_is too large to estimate in these cases, but its products with small numbers, viewed as single *K*_*j *_parameters, might still be estimable.

In the graphs shown in Figure 2, it is plausible to conjecture *a priori *that any two or all three of *K*_*d_R_R*_, *K*_*d_Rt_R *_and *K*_*d_Rt_Rt *_are equal, i.e. that the binding of t to R has no impact on R binding to itself. Similarly, it is plausible that any two or all three of *K*_*d_R_t*_, *K*_*d_RR_t *_and *K*_*d_RRt_t *_are equal. These two sets of hypotheses are not independent, since *K*_*d *_products of two paths between the same two nodes must be equal. For example, in Figure 2A, starting with free reactants, the two paths to RRt are

$$\begin{array}{c} K_{d\_R\_R\_t}=K_{d\_R\_R}K_{d\_RR\_t}\\ =\frac{\left[R][R\right]}{2[RR]}\frac{2[RR][t]}{\left[RRt\right]}=\frac{{\left[R\right]}^{2}[t]}{\left[RRt\right]}\\ K_{d\_R\_R\_t}=K_{d\_R\_t}K_{d\_Rt\_R}\\ =\frac{\left[R][t\right]}{\left[Rt\right]}\frac{\left[Rt][R\right]}{\left[RRt\right]}=\frac{{\left[R\right]}^{2}[t]}{\left[RRt\right]} \end{array}$$

and the two paths to RRtt are

$$\begin{array}{c} K_{d\_R\_R\_t\_t}=K_{d\_R\_R}K_{d\_RR\_t}K_{d\_RRt\_t}\\ =\frac{\left[R][R\right]}{2[RR]}\frac{2[RR][t]}{\left[RRt\right]}\frac{\left[RRt][t\right]}{2[RRtt]}\\ =\frac{{\left[R\right]}^{2}{\left[t\right]}^{2}}{2[RRt]}\\ K_{d\_R\_R\_t\_t}=K_{d\_R\_t}^{2}K_{d\_Rt\_Rt}\\ =\frac{\left[R][t\right]}{\left[Rt\right]}\frac{\left[R][t\right]}{\left[Rt\right]}\frac{\left[Rt][Rt\right]}{2[RRtt]}\\ =\frac{{\left[R\right]}^{2}[t]}{2[RRt]}. \end{array}$$

Similarly, the two paths from the node [Rt R t] to RRtt yield

$$\begin{array}{c} K_{d\_Rt\_R}K_{d\_RRt\_t}=\frac{\left[Rt][R\right]}{\left[RRt\right]}\frac{\left[RRt][t\right]}{2[RRtt]}\\ =\frac{\left[Rt][R][t\right]}{2[RRt]}\\ K_{d\_R\_t}K_{d\_Rt\_Rt}=\frac{\left[R][t\right]}{\left[Rt\right]}\frac{\left[Rt][Rt\right]}{2[RRtt]}\\ =\frac{\left[Rt][R][t\right]}{2[RRt]}, \end{array}$$

though these could have been obtained from (11) and (12). Based on Eqs. (11), either of *K*_*d_R_t *_= *K*_*d_RR_t *_and *K*_*d_R_R *_= *K*_*d_Rt_R *_implies the other, and based on Eqs. (13), either of *K*_*d_R_t *_= *K*_*d_RRt_t *_and *K*_*d_Rt_R *_= *K*_*d_Rt_Rt *_implies the other. Such constraints yield the *K*_*d *_equality hypotheses shown in Fig. 2. This space of *K*_*d *_equality models was generated from the fully constrained Model A by releasing pairs of R binding equality constraints and counterpart t binding constraints one at a time. When two R binding constraints are released, all three R binding constants become independent, and this leaves only one permissible t-binding constraint (Model E) or none (Model F). Models with one node less (G to N) are then considered; the two Rt nodes act as one. Models with two or more nodes removed do not allow *K*_*d *_equality constraints and in these cases, *K*_*j *_defined by Eq. 4 are adequate; such models are shown in Figure 3.

The Rt system full model special case of *g *= 0 in Eqs. (2), with *T*_*n *_= ([*R*_*T*_], [*t*_*T*_]), *F*_*n *_= ([*R*], [*t*]), *Z*_*n *_= ([*Rt*], [*RR*], [*RRt*], [*RRtt*]), and thus

$$W=\left(\begin{array}{cccc} 1 & 1 & 2 & 2\\ 1 & 0 & 1 & 2 \end{array}\right),$$

is

$$\begin{array}{lll} 0 & = & p[R_{T}]-[R]-\frac{\left[R][t\right]}{K_{Rt}}-2\frac{{\left[R\right]}^{2}}{K_{RR}}\\ & & -2\frac{{\left[R\right]}^{2}[t]}{K_{RRt}}-2\frac{{\left[R\right]}^{2}{\left[t\right]}^{2}}{K_{RRtt}}\\ 0 & = & [t_{T}]-[t]-\frac{\left[R][t\right]}{K_{Rt}}\\ & & -\frac{{\left[R\right]}^{2}[t]}{K_{RRt}}-2\frac{{\left[R\right]}^{2}{\left[t\right]}^{2}}{K_{RRtt}}. \end{array}$$

These *g *= 0 equations correspond to graph A in Figure 3. As *K*_*j *_= ∞ assumptions are applied to these equations to remove specific terms one at time, two at a time, and so on, corresponding nodes are removed from graph A to create graphs B to P and thus models that conjecture that the deleted nodes/complexes are not detectable above noise. Of these models, the *J *single edge models (L to O) can have additional *K*_*j *_= 0 assumptions applied to them to generate *J *additional *g *models (Q to T), each alleging that the free concentration of the reactant that is not in excess (i.e. ligand or R) is indistinguishable from zero (i.e. at a level too low to be detected using the data at hand). In such models, *K*_*j *_= 0 is handled either by approximating 0 by a small number (e.g. .0001; this option is readily automated, but pushing it too far causes numerical problems) or by replacing the equations with rules (e.g. if *K*_*RRtt *_= 0 as in Model 3R, the rule would be: if [*R*_*T*_] *<*[*t*_*T*_], [*R*] = 0 and [*RRtt*] = [*R*_*T*_]/2, else [*R*_*T*_] ≥ [*t*_*T*_] and thus [*R*] = [*R*_*T*_] - [*t*_*T*_] and [*RRtt*] = [*t*_*T*_]/2; this option remains to be automated). In the end, a spur graph (e.g. 3A) with *J *edges generates 2^*J *^models via *K*_*j *_= ∞ assumptions and an additional *J *models via *K*_*j *_= 0 assumptions, e.g. the 2^4 ^+ 4 = 20 models in Fig. 3. Considering that *J *is the number of complex species, which can be large, the number of *g *models generated can be huge.

The models in Figs. 2 and 3 are characterized by their assignments to the four *K*_*j *_parameters in Eq. 15 as shown in Table 1. This table defines a standard space of *K *hypothesis *g *models for ligand induced protein dimerization equilibria. As Models F, H, J, L and N in Fig. 2 do not have any *K*_*d *_equality constraints, their data fitting capabilities are equal to those of Models A through E in Fig. 3, respectively. To see this, consider the first of the two rows labeled 3A,2F in Table 1. Eqs. (5) and (8) give *K*_*RR *_= 2*K*_*d_R_R *_and *K*_*Rt *_= *K*_*d_R_t*_, Eq. (11) gives *K*_*RRt *_= *K*_*d_R_t*_*K*_*d_Rt_R*_, which can be adjusted independently by the factor *K*_*d_Rt_R*_, and Eq. (12) gives *K*_*RRtt *_= $2K_{d\_R\_t}^{2}K_{d\_Rt\_Rt}$*K*_*d_Rt_Rt*_, which can be adjusted independently by *K*_*d_Rt_Rt*_. Thus, all four of the *K*_*j *_parameters of 3A can be independently manipulated to arbitrary values by the four *K*_*d *_parameters of 2F, and in this sense, the two models are equivalent. A major difference, however, is that 2F can be represented in more than one way. Indeed, two choices are given by the two 3A,2F rows in Table 1, and all of the graphs in Figure 2 can be parameterized as subsets of either the E-shaped or ⊓-shaped parameterization topologies given in these two full model rows.

The nine grid graphs in Fig. 2 that contain at least one *K*_*d *_= ${K^{\prime }}_{d}$ constraint have *|K*_*j*_*| > |K*_*d*_| where *|K*_*x*_| is the number of freely estimated *K*_*x *_parameters. Meanwhile, models that are equally well represented by both grid and spur graphs are characterized by *|K*_*j*_| = *|K*_*d*_|, which, in Fig. 3, is all of the graphs except I, J, M, N, R and S. These exceptions must use spur graphs to avoid non-identifiability problems, have *|K*_*j*_*| < |K*_*d*_|, include complexes without including required intermediates, and have *K*_*d *_= ∞ in product expressions that remain finite (see Table 1 footnote). Such models are palatable only because they represent statistical null hypotheses rather than physical null hypotheses, i.e. *K*_*d *_= ∞ is a claim that the true value of *K*_*d *_is too large to estimate based on the data at hand, and not a claim that binding never occurs.

### *p *hypotheses

The probability that an R molecule is undamaged can be hypothesized to be close enough to 1 that the data cannot discriminate it from being 1. If *B *different protein preparation batches (indexed by *b*) are used in the experiments, 2^*B *^hypotheses exist. *p*_*b *_= *p*_*b*' _hypotheses that two batches are equivalent can also be formulated. In the equations given above and in the data analysis given below, *B=1* is assumed.

### Measurement models *h*

In pairs (*g*, *h*) the system of interest *g *is separated from the methods used to study it in *h*. *h *maps steady states *F*_*n *_of *g *into expected values of measurements E(*y*_*n*_). The first step in this, common to all *h *models, is to convert the *F*_*n *_into complex concentration predictions *Z*_*n *_using Eq. (1), i.e. using *W *and *K*. The second step is to form E(*y*_*n*_) from *F*_*n *_and *Z*_*n *_and any other available information (e.g. *L *and *p*; note that *T*_*n *_can be reconstructed from *F*_*n *_and *Z*_*n*_). This second step is different for different measurement types, as illustrated below for average protein mass, fraction of protein bound to a particular ligand, and average enzymatic activity of a distribution of enzyme states.

#### average mass

Suppose R is the only protein in the system, that ligand masses are too small to be detected relative to protein masses, and that average protein mass measurements are mass-weighted, e.g. as in dynamic light scattering data [1-3]. The second step of *h *for this type of measurement is then

$$E(y_{n})=M_{1}\frac{[R]+[R_{T}](1-p_{b})+\sum_{j=1}^{J}Z_{nj}W_{1j}^{2}}{\left[R_{T}\right]}$$

where *M*_1 _is the mass of R monomer.

#### fraction bound

For fraction of protein bound to ligand data, suppose the ligand of interest is the *i*th reactant. The fraction of R bound to ligand is then

$$E(y_{n})=\left(\sum_{j=1}^{J}Z_{nj}W_{ij}\right)/[R_{T}].$$

#### enzyme activity

If *k*_*catj *_is the per-active-site enzymatic activity of the *j*th complex, the measured average activity of an ensemble of complexes is

$$E(y_{n})=\left(\sum_{j=1}^{J}k_{catj}Z_{nj}W_{1j}\right)/[R_{T}].$$

It is assumed here that R provides all of the enzymatic activity and that it has only one active site.

### *h *space

Enzyme activity differs from the other two measurement types in that its parameters can have many plausible null hypotheses: the *k*_*catj *_can be equal to zero or to each other within groups defined in various ways. Thus, Eq. (18) can generate a space of *h *models. When such a space is multiplied into a *g *space, not all *h *models can be paired with any *g*, since, for example, if a *K*_*j *_is infinity in a *g *model, the corresponding product complex concentration is zero, so a corresponding *k*_*cat *_cannot be estimated. Thus, although to first order |(*g*, *h*)| = *|g||h| *where *|x| *is the number of *x *models, this is actually an upper bound.

### dTTP induced R1 dimerization data analysis

**Figure 4:**
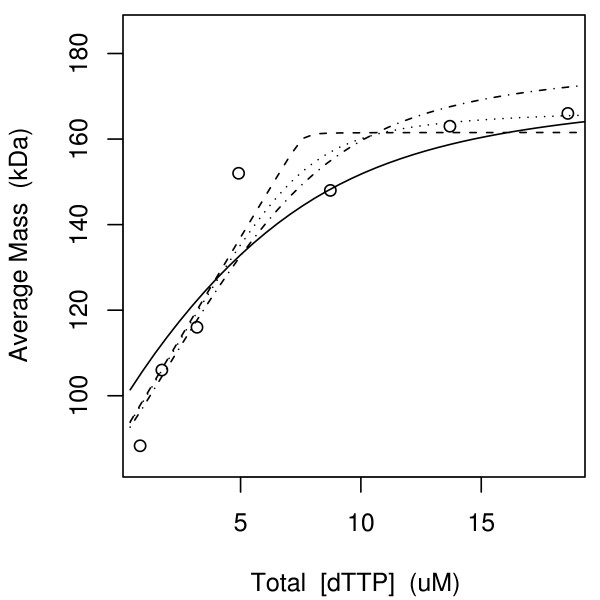
**Scott et al. data**. The parameter values of Scott et al. (Table 2, initial values of Model 2E) do not fit the data well (solid curve). The same model with fully converged parameter values does fit the data well (dotted). With *p *freely estimated, the infinitely tight binding Model 3Rp (dashed) has the lowest AIC. The second lowest AIC was achieved by Model 3M (dashed-dotted), see Table 2.

Let R be the R1 subunit of ribonucleotide reductase and let t be dTTP. Using *h *in Eq. (16), Scott et al [1] fitted Model 2E with *p *= 1 to their dynamic light scattering data shown in Figure 4. Their final parameter estimates are shown as the initial parameter estimates in Table 2. That these estimates did not converge properly (the authors used a method similar to that of Storer and Cornish-Bowden [7] to solve their *g *= 0 equations) is evidenced by the poor fit of the solid curve in Figure 4 relative to its fully converged counterpart computed here using the *g *= 0 solver described above (Eq. 3; dotted curve). The consequences of this poor fit are seen to be substantial in Table 2, where many of the *K*_*d *_estimates have initial values that differ from their final converged counterparts by an order of magnitude. The final *K*_*d *_estimates are, however, very uncertain, with upper-to-lower 95% confidence interval (CI, see Methods) limit ratios of ~10^6^, i.e. Model 2E is overparameterized.

**Table 2:** Parameter estimates corresponding to Figure 4

Model	Parameter	Initial Value	Optimal Value	Confidence Interval
3Rp	pRT	1.000	0.767	(0.662,0.890)
	Rt	Inf	Inf	absent
	RR	Inf	Inf	absent
	RRt	Inf	Inf	absent
	RRtt	0.000	0.000	fixed
	SSE	0.100	0.027	
	AIC	-26.948	-36.058	
	cpu	0.000	0.057	fit succeeded
3M	RRtt	1.000	17.231	(3.190,93.691)
	Rt	Inf	Inf	absent
	RR	Inf	Inf	absent
	RRt	Inf	Inf	absent
	pRT	1.000	1.000	fixed
	SSE	0.059	0.032	
	AIC	-30.657	-34.969	
	cpu	0.000	0.291	fit succeeded
3Mp	RRtt	1.000	1.838	(0.010,347.234)
	pRT	1.000	0.837	(0.656,1.067)
	Rt	Inf	Inf	absent
	RR	Inf	Inf	absent
	RRt	Inf	Inf	absent
	SSE	0.059	0.023	
	AIC	-26.457	-32.981	
	cpu	0.000	0.109	fit succeeded
3N	RRt	1.000	16.699	(6.821,40.854)
	Rt	Inf	Inf	absent
	RR	Inf	Inf	absent
	RRtt	Inf	Inf	absent
	pRT	1.000	1.000	fixed
	SSE	0.176	0.045	
	AIC	-22.987	-32.602	
	cpu	0.000	0.125	fit succeeded
2E	R_t	25.000	2.265	(0.004,1164.445)
	R_R	75.000	1451.803	(0.089,24154952.754)
	RR_t	0.550	0.024	(0.000,22.421)
	RRt_t	0.550	0.024	constrained
	pRT	1.000	1.000	fixed
	SSE	0.042	0.027	
	AIC	-21.806	-24.990	
	cpu	0.000	0.264	fit succeeded

Given knowledge that R has a binding site for t and that R can dimerize [12], the model space in Table 1 doubled by *p *free or fixed to 1 and coupled to *h *in Eq. 16 creates 58 (*g*, *h*) candidate models that were fitted to these data. The fitted models were ranked by the Akaike Information Criterion (AIC, see Methods) and the best model was 3Rp (*p *freely estimated) with *K*_*RRtt *_= .0001 *μ*M^3 ^essentially fixed to zero (dashed straight lines in Figure 4; Table 2). This model represents a tight binding titration limit wherein free molecule annihilation (the initial linear ramp in Fig. 4) continues in a one-to-one fashion with increasing [dTTP_*T*_] until [dTTP_*T*_] equals [R1_*T*_] = 7.6 *μ*M, the plateau point beyond which all dimerizable R has dimerized. The second best model (dashed-dotted in Figure 4) was 3M (*p *fixed to 1) with *K*_*RRtt *_freely estimated as 17 *μ*M^3^. This second best model is the best model when recent gel filtration data [4] shown in Table 3 are also included in the analysis, see Table 4 (2E ranked 20th and 13th in Tables 2 and 4 in exhaustive model space fits and was not even fitted by the semi-exhaustive method described next).

**Table 3:** Rofougaran et al.'s R1 dimerization data

R_*T*_	t_*T*_	Dimer	Monomer	Average Mass
2.700	100	18100	910	175.692
0.135	100	693	98	168.850
2.700	0	935	19766	94.065

**Table 4:** Joint Data Analysis

Model	Parameter	Initial Value	Optimal Value	Confidence Interval
3M	RRtt	1.000	18.697	(4.807,72.966)
	Rt	Inf	Inf	absent
	RR	Inf	Inf	absent
	RRt	Inf	Inf	absent
	pRT	1.000	1.000	fixed
	SSE	0.064	0.034	
	AIC	-48.066	-54.448	
	cpu	0.000	0.445	fit succeeded
3Mp	RRtt	1.000	5.558	(0.370,83.931)
	pRT	1.000	0.907	(0.787,1.044)
	Rt	Inf	Inf	absent
	RR	Inf	Inf	absent
	RRt	Inf	Inf	absent
	SSE	0.064	0.027	
	AIC	-44.852	-53.308	
	cpu	0.000	0.199	fit succeeded
3Rp	pRT	1.000	0.822	(0.736,0.918)
	Rt	Inf	Inf	absent
	RR	Inf	Inf	absent
	RRt	Inf	Inf	absent
	RRtt	0.000	0.000	fixed
	SSE	0.106	0.041	
	AIC	-42.954	-52.590	
	cpu	0.000	0.104	fit succeeded
3I	RRt	1.000	49.568	(5.755,428.375)
	RRtt	1.000	37.930	(5.003,290.035)
	Rt	Inf	Inf	absent
	RR	Inf	Inf	absent
	pRT	1.000	1.000	fixed
	SSE	0.165	0.030	
	AIC	-35.303	-52.218	
	cpu	0.000	0.223	fit succeeded
2E	R_t	25.000	143.621	(0.477,44355.855)
	R_R	75.000	956.076	(0.790,1202604.284)
	RR_t	0.550	0.106	(0.001,8.085)
	RRt_t	0.550	0.106	constrained
	pRT	1.000	1.000	fixed
	SSE	0.079	0.031	
	AIC	-38.357	-47.750	
	cpu	0.000	0.344	fit succeeded

### Semi-exhaustive model selection

The semi-exhaustive model selection algorithm is: (1) create a list of all of the candidate models; (2) sort it according to the number of freely estimated parameters in each model; (3) fit all of the models with the fewest number of parameters; (4) fit all models with one additional parameter; and (5), repeat step 4 as long as the current batch of models has an improved AIC relative to the previous batch of models. In the case of the Rt system, compared to exhaustive fits to the entire space of 58 (*g*, *h*) models, this algorithm stops before fitting the most time consuming over-parameterized models (those with three parameters or higher) though it identifies the exact same top 13 (Table 2) and top 7 (Table 4) models. CPU times to compute Tables 2 and 4, expressed as exhaustive to semi-exhaustive ratios, averaged 4.7 (4.3/.89, 5.8/1.25, in minutes/minutes) when using 4 CPUs and 5.9 (14.8/2.5, 20.3/3.5) when using 1 CPU, or, rewritten, quad processor gains averaged 3.5 (14.8/4.3, 20.3/5.8) for exhaustive fits and 2.8 (2.5/.89, 3.5/1.25) for semi-exhaustive fits, i.e. there are semi-exhaustive approach losses in parallel processing efficiency as some CPUs become idle while the last models in a batch are fitted.

## Implementation

R codes are provided to insure reproducibility of the results. They are also provided because they may be useful in other ligand induced protein dimerization data analyses. The following script illustrates their use.

setwd("/home/radivot/case/active/rnr/Rt/R")

load("RNR.RData") # load RNR adata

source("fRt.r") # function definitions

# the next line generates and compiles C code

g=mkgObj("Rt", c("Rt","RR","RRt","RRtt"))

RtData=adata [c("f1a01")] # Scott et al 2001 Rt data

# these map Kd into Kj as shown in Table 1

Eshape<-function(x)

   c(x[1], 2*x[2], x[1]*x[3], 2*x[1]^2*x[4])

nshape<-function(x)

   c(x[1], 2*x[2], x[2]*x[3], 2*x[2]*x[3]*x[4])

models=list(

mkModelObj(RtData, g, "2E",

   Kdparams=c(R_t=30, R_R=85, RR_t=.55, RRt_t=.55),

   Keq=c(RRt_t="RR_t"), Kd2Kj=nshape),

mkModelObj(RtData, g, "3Rp",

   Kjparams=c(Rt=Inf, RR=Inf, RRt=Inf, RRtt=0),

   pparams=c(pRT=1)),

mkModelObj(RtData, g, "3M",

   Kjparams=c(Rt=Inf, RR=Inf, RRt=Inf, RRtt=1))

)

fitMS(models,"MS2")

In this script, load loads the RNR data provided in Additional File 1 and source reads in the function definitions provided in Additional File 2. The main function, fitMS, fits the model space (2E, 3Rp, 3M) and writes the results to html and LaTeX files. It can be passed options to specify the number of CPUs and the choice of semi-exhaustive or exhaustive fitting. A script that fits all 58 (*g*, *h*) models is provided as Additional File 3.

## Discussion

The most common approach to modeling is to manually identify several plausible models, fit them all, and accept the best in the lot, e.g. [13,14]. This approach works because human intuition carries external information that guides the choice of the initial lot. If the best model does not provide a good fit, or if it has parameters with very large confidence intervals, the lot can be augmented to include additional models with more or fewer parameters, respectively. The advantage of this approach is that only a handful of models needs to be fitted. The disadvantage is that different analysts can yield different results. In general, a model/hypothesis (e.g. that the experimental data cannot discriminate some *K*_*j *_from ∞ or zero, or that some *K*_*d *_equal others) is rejected if it is not among the best models selected, and supported if it is. Although inferences made from any model, including the best models, are always conditional on the truth of the model's assumptions, the likelihood of this truth increases as the model withstands elimination. This statement is valid only to the extent that alternative hypotheses are represented in the model space. For example, if a *K*_*d *_= ${K^{\prime }}_{d}$ model assumes symmetric oligomers (e.g. as in Eqs. 9 and 10) and the model space does not include counterpart models that assume asymmetric forms, the selection process can lend no additional support to the symmetry assumptions. On the other hand, if independent data support such symmetry assumptions, the use of a restricted model space may be acceptable. It is anticipated that large model spaces will generate many models that are roughly equally best. Overall inferences should then reflect an average of the inferences of the best models, perhaps weighted by some metric of closeness to the optimum. Methods of accomplishing this for (*g*, *h*) models is an important area of future work. Another important area is automated model space enumeration: although this can be readily achieved for *K*_*d *_= ∞ or 0 spur graphs, it remains a challenge to achieve this for *K*_*d *_= ${K^{\prime }}_{d}$ grid graphs.

## Conclusion

The process of extracting *K *estimates from data is inseparable from the process of (*g*, *f*) model selection. This process requires clear statements of the model space explored, the criterion used to rank models, and the method used to search the space. If standards can be developed for these entities, analyst-to-analyst variations in inferences made from identical datasets could be reduced.

## Methods

### Data procurement

Plot Digitizer [15] was used to digitize the data of Scot et al. shown in Fig. 4. These data were originally given with model-dependent free concentrations on the *x*-axis. Such *x *values were converted to total concentrations using the model and parameter values given by Scot et al. [1]. The data in Table 3 is from Fig. 1 of [4]. It was kindly provided by Dr. Anders Hofer.

Model selection

With *P *equal to the number of freely estimated model parameters, *N *equal to the number of steady state data points, and *SSE *equal to the sum of squared errors of the fitted model, the Akaike Information Criterion [16] used here has the form *AIC *= 2*P *+ *N *log(*SSE/N*) + $\frac{2P(P+1)}{N-P-1}$[17]. This explicit metric states how much goodness of fit (*SSE*) one is willing to sacrifice to gain the benefit of one less parameter. For a given model, *P *and *N *are fixed, so *AIC *minimization reduces to *SSE *minimization by least squares.

### Parameter estimation

Best fitting SSEs were found by nonlinear least squares using the optim function in R [11] with the Nelder-Mead [18] option for *P > *1, the BFGS option for *P *= 1, and the Hessian option set to TRUE (see Additional Files). Hessians of the *SSE*s evaluated at the optimum were divided by 2, inverted, and multiplied by the mean squared error, *MSE *= *SSE/*(*N *- *P*), to compute parameter estimate covariance matrices. From these, parameter estimate standard deviations were taken as the square roots of the main diagonal, and these were then multiplied by 1.96 to approximate 95% CIs. All parameters were estimated as *e*^*c *^to constrain point estimates and CIs to positive values.

## Authors' contributions

TR performed all of the work and wrote the manuscript.

## Supplementary Material

Additional File 1 RNR.RData **= Data file** Click here for file

Additional File 2 fRt.r **= R function definitions** Click here for file

Additional File 3 Rt.r **= R script used** Click here for file
